# Release and Detection of microRNA by Combining Magnetic Hyperthermia and Electrochemistry Modules on a Microfluidic Chip

**DOI:** 10.3390/s21010185

**Published:** 2020-12-29

**Authors:** Marie-Charlotte Horny, Vincent Dupuis, Jean-Michel Siaugue, Jean Gamby

**Affiliations:** 1 Université Paris-Saclay, CNRS, Centre de Nanosciences et de Nanotechnologies, 91120 Palaiseau, France; mcha.horny@gmail.com; 2 Sorbonne Université, CNRS, Physico-Chimie des Électrolytes et Nanosystèmes Interfaciaux, PHENIX, F-75005 Paris, France; vincent.dupuis@sorbonne-universite.fr (V.D.); jean-michel.siaugue@sorbonne-universite.fr (J.-M.S.); 3 Sorbonne Université, CNRS, Laboratoire Interfaces et Systèmes Electrochimiques, LISE, F-75005 Paris, France

**Keywords:** microfluidics, nucleic acids, channel microelectrode, magnetic hyperthermia, magnetic release, core–shell nanoparticles, amorphous carbon nitride, early diagnostics

## Abstract

The heating of a biologic solution is a crucial part in an amplification process such as the catalytic detection of a biological target. However, in many situations, heating must be limited in microfluidic devices, as high temperatures can cause the denaturation of the chip components. Local heating through magnetic hyperthermia on magnetic nano-objects has opened the doors to numerous improvements, such as for oncology where a reduced heating allows the synergy of chemotherapy and thermotherapy. Here we report on the design and implementation of a lab on chip without global heating of samples. It takes advantage of the extreme efficiency of DNA-modified superparamagnetic core–shell nanoparticles to capture complementary sequences (microRNA-target), uses magnetic hyperthermia to locally release these targets, and detects them through electrochemical techniques using ultra-sensitive channel DNA-modified ultramicroelectrodes. The combination of magnetic hyperthermia and microfluidics coupled with on-chip electrochemistry opens the way to a drastic reduction in the time devoted to the steps of extraction, amplification and nucleic acids detection. The originality comes from the design and microfabrication of the microfluidic chip suitable to its insertion in the millimetric gap of toric inductance with a ferrite core.

## 1. Introduction

Within the framework of early diagnosis, decreased time scales lead to more efficient treatments but also to less overwhelmed health care centers. With a lower cost per test, molecular diagnosis could be used more often as routine liquid biopsy tests, making it an alternative to tissue biopsy or medical imaging such as magnetic resonance imaging (MRI). Currently, sensitivity, specificity and time scales are key parameters for efficient assays from sample to result. Multiple molecular steps are involved in molecular diagnostics to ensure result accuracy, but three of them step out as crucially needing reduction because of their time scales: target extraction, target release and target detection.

To this goal, miniaturization and coupling of various processes of molecular biology on the same device were reported to decrease analysis time, to reduce the volume of reagents and to decrease contamination with fewer steps requiring an operator [1,2,3]. The most advanced devices that meet these specifications are using PCR amplification (polymerase chain reaction) coupled with microfluidics. For each step and amplification cycle (90 °C, 55 °C, 70 °C and 37 °C), the temperature has to be precisely controlled over a large range [4], which is time-consuming, and absolute quantification cannot be reached especially for short RNA sequences [5]. To overcome these limitations, some recent innovations, digital PCR in droplets, [6,7,8,9], isothermal PCR (loop-mediated amplification—LAMP, [10] nucleic acid sequence-based amplification—NASBA and [11] rolling circle amplification—RCA, [12,13]) are becoming interesting alternatives to the classical PCR. Houssin et al. [14] have demonstrated a molecular amplification with a non-isothermal chip with an ultra-rapid thermalization speed using the temperature controller Cherry Biotech (heat cooler for microscopy in the 5–70 °C temperature range) [10].

Closely following on-chip PCR progresses, recent advances in on-chip electrochemistry have been obtained due to standard photolithographic or laser photoablation technologies and microelectrodes to fit into microfluidic devices [15,16,17,18,19,20,21,22]. Electrochemistry has a major advantage over other detection methods, in that it directly transduces biological events (e.g., DNA hybridization) without any further transformation of the physical signal [23,24]. Combining high specificity, cheapness, ease of use, portability and compatibility with microfabrication processes, electrochemical biosensors are excellent candidates for clinical interest diagnosis. During the DNA hybridization step, the target’s sequence is identified with a DNA probe that possesses a complementary sequence to the one of the target DNA (Watson and Crick’s rules [25]). Along this line, Ferguson et al. [4] reported an integrated microfluidic electrochemical DNA (IMED) sensor coupling two modules on chip e.g., PCR and electrochemical detection (ED) with an enzyme digestion in between to get a single-strand DNA. The PDMS/glass chip is composed of a PCR chamber that performs 38 cycles of amplifications in 1 h 40 min (including enzyme digestion), controlled by a temperature controller regulating a platinum resistive temperature detector mounted onto a custom thermofoil pad. Their gold transducer is composed of a mixed probe layer with thiolates adsorbed and a methylene blue redox labeled probe diluted with 6-mercapto-1-hexanol. Their device demonstrated a 10 attomolar LOD (limit of detection) for non-purified genomic DNA (17-basis).

All the above described coupling technologies must have a heating system with a thermalization chamber and a feedback loop for temperature, whether it is a heating via Joule effect (nanoheaters), Peltier, infrared or microwaves [13,26,27]. For these reasons, local heating in or outside a chip is an attractive alternative to the global heating of a biological sample.

Local heating using magnetic fluid hyperthermia on magnetic nanoparticles has proven to induce the death of tumor cells [28,29,30,31], and the release of DNA targets attached to their probe and immobilized on magnetic nanoparticles [32,33]. The use of a complex external temperature module with complex calibration systems between water inset, heating induced by imaging devices and temperature compensation is avoided. This makes it possible to use a heater with a temperature range from 0 to 95 °C, without a feedback loop and direct temperature measurement of the sample. Dias et al. [32] showed, using DNA strands of different length and hyperthermia, that the rise in local temperature can be recorded. Similarly, Dong et al. [34] assessed the temperature in the vicinity of nanoparticles.

Here we report on magnetic hyperthermia transposition in microfluidics coupled to electrochemistry. The latter opens the way to a drastic reduction in the time devoted to microRNA targets extraction, amplification and detection steps. The major originality of our work comes from the microfluidic device design and microfabrication adapted to its insertion into the millimetric gap of toric inductance with a ferrite core.

In the first section, the proof of concept of microRNA release with on-chip magnetic hyperthermia at room temperature is presented. Hyperthermia in microfluidics allows a fine and dynamic tuning of a confined environment (concentrations, flow rates and time of residency of particles), while keeping the volume of reaction under the microliter, especially relevant for the use of expensive biological samples. The second section is dedicated to microelectrode integration on the on-chip magnetic hyperthermia device to combine microRNA release and electrochemical detection in a one-step microfluidic protocol.

## 2. Materials and Methods

### 2.1. Chemicals

Sodium chloride (NormaPur), methylene blue (Alfa Aesar), potassium ferricyanide (III), and potassium hexacyanoferrate (II) (Sigma-Aldrich, St Quentin Fallavier, France) were used in the experiments without further purification. N-(3-Dimethylaminopropyl)-N-ethylcarbodiimide hydrochloride (EDC); N-Hydroxy-sulfosuccinimide sodium salt (NHS); 3-(N-Morpholino)propanesulfonic acid sodium salt (MOPS) and tetraethoxyorthosilicate (TEOS); 3-(aminopropyl)triethoxysilane (APTS) were purchased from Sigma–Aldrich (St Quentin Fallavier, France). Citric acid was purchased from Merck (Nogent sur Marne, France). 2-[Methoxy(polyethyleneoxypropyl]-trimethoxysilane (PEOS), containing 3–6 ethylene oxide groups, was purchased from Gelest (Morrisville, PA, USA). From VWR (Strasbourg, France) 30% H_2_O_2_, HCl and NaOH solutions were obtained.

### 2.2. Oligonucleotide Sequences

The oligonucleotides were purchased from Integrated DNA Technology (Belgium). The DNA probe (P) is a 21-basis long nucleic acid and is modified with a carboxy-end for conjugation to magnetic nanoparticles. The DNA probe (P’) is modified with an amino-end for microelectrode functionalization. The target is an unmodified DNA sequence of 20 bases as written in Table 1 mimicking the fragment of interest, miRNA-122, for the diagnosis of liver cells in case of injury (hepatitis, alcoholism and obesity).

### 2.3. DNA Hybridization on Core–Shell Nanoparticles γ-Fe_2_O_3_ @SiO_2_ PEG/NH_2_, Release and Target Detection

Core–shell nanoparticles γ-Fe_2_O_3_@SiO_2_ PEG/NH_2_ were synthetized (see Appendix A) and functionalized with the carboxy-modified DNA probe (Table 1). An amount of 120 µL of a carboxy-modified DNA probe at 1000 µg mL^−1^ and 120 µL of a complementary DNA target (10:1 probe) at 1 × 10^4^ µg mL^−1^ were left to hybridize in MOPS 0.1 M pH 7.4, NaCl 0.5 M for 30 min. Coupling reagents EDC and NHS (200 and 335 equivalents related to the DNA-probe respectively) were poured into the reacting solution for 20 min. A volume of 78 µL of core shell nanoparticles solution ([Fe] = 0.016 M) was added and left to react overnight. Miltenyi columns were used for magnetic separation, performed by rinsing twice with 500 µL of MOPS 0.1 M pH 7.4 and NaCl 0.5 M followed by DNA grafted core shell nanoparticles elution with 1 mL of MOPS 0.1 M, pH 7.4 and NaCl 0.5 M.

After DNA duplex immobilization on magnetic nanoparticles, the duplexes were introduced in the microfluidic device at a specific flow rate with the use of a programmable syringe pump. For the fluorescence measurements, either 100 or 400 µL of the outlet solution from the device was collected and filtrated with a Miltenyi column to discard nanoparticles before DNA target released for measurement.

### 2.4. Fluorometric Measurements Calibration

All DNA sequences quantification measurements were done using fluorescence spectroscopy performed, in cuvettes, on a Cary Eclipse fluorometer (λ_excitation_ = 480 nm, λ_emission_ = 520 nm) with the Quant-iT™ Oligreen ss-DNA Assay Kit from Invitrogen Thermo Fisher Scientific (Villebon sur Yvette, France). Calibration curve of Quant-iT™ Oligreen was realized with standards of our own micro-RNA for the target calibration.

### 2.5. Electrochemical Measurements Protocol

#### 2.5.1. Microelectrode Activation Protocol

The a-CN_0.12_ as-grown electrodes show poor reactivity and require an electrochemically pretreatment to improve their surface reactivity [35,36]. Anodic activation in 0.1 M KOH was carried out to favor both reactivity and an increase of oxygen dangling bonds (under the form of carboxyl groups) at the carbon surface. This step is crucial to future immobilization of amino-modified DNA strands (NH_2_-DNA) on aCN_0.12_. It consists of an introduction of a 0.1 M KOH solution in the microfluidic channel with a 0.5 µL s^−1^ flow rate and a 2.7 × 10^−5^ mA applied galvanostatic current for the anodic activation. The carboxyl groups dangling bonds on the working microelectrodes (WE) were previously activated (due to an EDC/NHS two-step protocol) before the functionalization with DNA-NH_2_ probes (1 mg mL^−1^). The a-CNx electrodes were screened in our two-electrode setup (30 µm a-CNx working electrode and 2 mm platinum counter electrode) in an equimolar (3 mM) mixture of Fe(CN)_6_^4−^/Fe(CN)_6_^3−−^ in NaCl (0.5 M) with MB (10^−8^ M). The microfluidic devices were connected with a potentiostat (Gamry 600 + apparatus) for electrochemical impedance spectroscopy (EIS) data acquisitions.

#### 2.5.2. Microelectrode Functionalization Protocol

Single stranded DNA probe immobilization in a microfluidic device was performed via a two-step protocol. First carboxyl groups on the a-CNx surface were activated by pouring a 2.10^−7^ M EDC and NHS solution inside the chip for 20 min. Then a 0.15 µM amino-modified DNA probe (see Table 1) was circulated in 0.5 M NaCl, after which the flow was stopped for three hours. The flushing was done with deionized water and the test of stability of the SAM was done in 0.5 M NaCl and 0.5 µL s^−1^ flow for 30 min.

### 2.6. Micro Nanofabrication Procedures

#### 2.6.1. Microfluidic Chip Fabrication for On-Chip Hyperthermia

**Figure 1:**
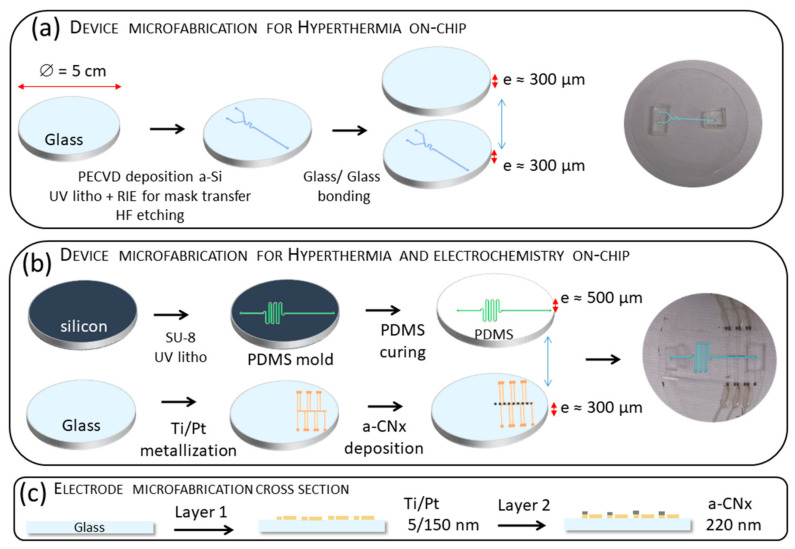
Schematic view of microfabrication key steps. (**a**) PDMS fabrication and bonding for on-chip hyperthermia. (**b**) Inverted optical lithography, Ti/Pt metallization and amorphous carbon nitride (aCNx) deposition by magnetron sputtering for on-chip electrochemistry. (**c**) Schematic cross section view of material layers deposition Ti/ Pt and a-CNx for counter-electrodes (CE) and working microelectrode (WE) networks, respectively. See Appendix B for more details on electrodes composition and design.

**Figure A3:**
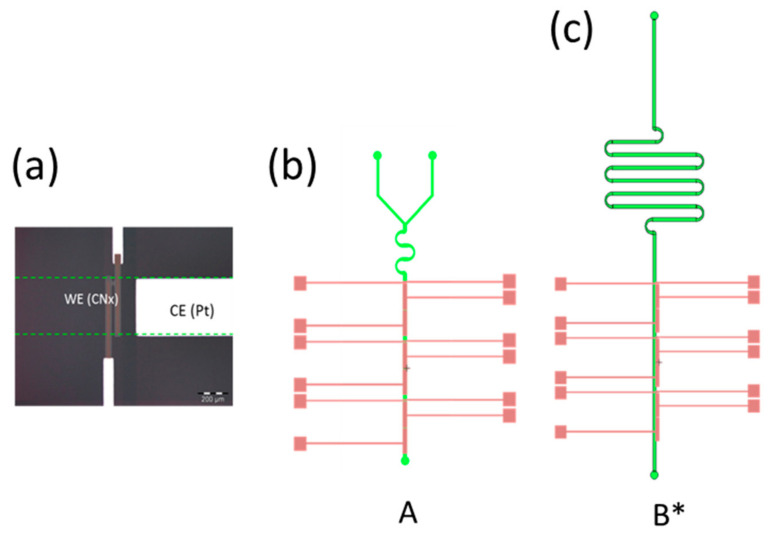
Photography and L-Edit channel and microelectrodes geometries. (**a**) Microscopic view of the working microelectrodes in amorphous nitride carbon (WE) on top of the counter electrode (CE) in platinum. (**b**) Fluidic channel with geometry A. (**c**) Fluidic channel with geometry B*. See dimensions on enlarged zone WE/CE electrode pair (Figure 2c,d) where WE stands for *a*-CNx working electrode (width, w = 300 μm, length, x_e_ = 30 μm), CE stands for platinum counter electrode (w = 300 μm, x_e_ = 2 mm) and the fluidic channel height and width are equal to 40 µm and 300 µm, respectively. Note that here channel and electrode widths are the same. The total effective volume of the sample depends on the microchannel volume (detection zone area, which is the same on A and B* configuration) is approximately equal to 0.24 µL covering the electrode pairs.

The glass/glass setup bearing only the fluidic channel includes a lower glass part with a 50 µm high channel etched in the glass (Figure 1a). First, glass was cleaned with H_2_O_2_/H_2_SO_4_ (1:1) prior to a plasma enhanced chemical vapor deposition (PECVD) deposit (20 sccm SiH_4_, 10 W, 40 min) of a thick, 600 nm layer of amorphous silicium (aSi), the mask for the etching. The amorphous silica (aSi) deposit was followed by a 400 °C annealing for 4 h to decrease mechanical constraints (notching effects). UV photolithography with AZ-5214 was used to draw the mask pattern. Two different channel geometries were used (see Appendix B, Figure A3). The pattern was transferred to the aSi layer with reactive ion etching (RIE, 10 W RF, 10 mTorr, 10 sccm SF6, 2 min 30). Finally the obtained substrate was exposed to a solution with a low HF dilution (HF/HCl/H_2_O, 10:3:20, 27 min). HCl addition in the mixture allows reduction of the microchannel’s bottom roughness according to Iliescu et al. [37]. The upper glass part acts as cover bounded hermetically to the lower part (glass/glass bounding patent [38]). The total thickness of the device does not exceed 600 µm.

#### 2.6.2. Microfluidic Chip Fabrication for On-Chip Hyperthermia and Electrochemistry

The chip is composed of two layers: a flat glass substrate bearing microelectrodes and a thin PDMS layer bearing the microfluidic channel serpentine as cover. The final device is obtained by bonding the upper PDMS part to the lower cleaned glass part (Figure 1b).

Briefly, the PDMS channels were made from a channel master mold made of SU8 on a Si wafer. The master mold was manufactured by spin-coating (30 s, 1500 rpm s^−1^, 200 rpm s^−2^). The photoresist SU8-2002 was used as an adhesive layer (MicroChem Corp, Westborough, MA, USA). After spin coating, the wafer was baked-up on a hot plate (65 °C for 3 min, then 95 °C for 5 min). At the end, a long baking was performed at 110 °C for 2 h. A 16 µm thick layer of SU8-2050 was spin-coated (30 s, 3700 rpm s^−1^, 200 rpm s^−2^). The photoresist was baked-up on a hot plate (65 °C for 3 min, then 95 °C for 5 min), exposed to UV light (25 s, 16.5 mW), developed using SU8 developer for 120 s and baked-up on a hot plate (65 °C for 2 min, then 95 °C for 7 min). The PDMS was fabricated by mixing silicon elastomer base and silicon elastomer curing agent in a 10:1 proportion. A few drops of PDMS were poured onto the SU8 master mold to obtain a 500 µm thick negative replica after degassing and curing the fluidic network at 70 °C overnight. The fluidic part was detached from SU8 mold with a scalpel. The holes for the inlets and outlet tube (0.75 mm diameter) were made out of a 1 cm thick PDMS and perforated by a punch hole. The PDMS and the glass substrates were washed with isopropanol and dried with nitrogen.

## 3. Results

### 3.1. Setup for Performing Magnetic Hyperthermia On-Chip

Microfluidic chip fabrication for hyperthermia (Figure 1a) and its coupling with on-chip electrochemistry (Figure 1b) are above described. Our miniaturized setup was inspired from the large scale setup from Lacroix et al. [39] where homogeneous radiofrequency (RF) magnetic field is produced inside a large 1 cm ferrite gap (in which the Eppendorf tube is inserted). In our homemade set-up, the homogeneous RF magnetic field is produced inside a small 1 mm gap (in which the microfluidic serpentine is inserted), cut in a low loss ferrite ring on which 55 turns of Litz wire are wound. This magnetizing coil is coupled to a 1 nF capacitor to form a series LC circuit, whose resonance frequency is about 180 kHz. The resonant circuit is excited at the resonance frequency by a signal generator coupled to a RF power amplifier and produces magnetic field amplitude of 370 Oe (measured using a single turn pick-up coil). The microfluidic channel was connected to a programmable syringe pump allowing flow rates from 0.01 to 5 µL s^−1^.

#### 3.1.1. Theory of Electromagnetism, an Electromagnet with a Gap

The setup is taking advantage of the use of an electromagnet with a millimetric gap (ca. 1 mm) with a winding composed of *n* spires in which is flowing a current *i*. The gap can be considered as a thin layer of air perpendicular to the median line of the circuit, and with a thickness far below the mean torus perimeter. In these conditions, edge effects can be neglected, and it is commonly admitted that the magnetic lines are parallel to the median line of the torus.

Assuming that the gap length is small (compared to the core length) and that the relative magnetic permeability of the ferrite is large, the field, *H*_e_, in the gap, e, can be written as follows

(1) $$\mathrm{He}\;=\;\frac{n\times i}{e}$$

with *n*, the number of spires, *i* the current intensity in the spires. The magnetic flux, ϕ, transiting through the circuit, especially in the gap, is given by:

(2) $$\varphi \;=\;\frac{\mu_{0}\times n\times i\times S}{e}$$

with $\mu_{0}$ represents the vacuum permeability and *S*, the spires’ surface.

Equation (2) highlights the possibility to reach a high field by focusing, in a small gap, the field produced by an inductance with dimensions far above the one of the gap [40].

#### 3.1.2. Magnetic Hyperthermia, Neel and Brown Contributions

Superparamagnetic particles exhibit a heating behavior when submitted to an alternative magnetic field. The calculation of magnetization relaxation times depends on the way the particle is rotating in the solvent of dispersion, either external (Brown relaxation) or internal (Neel relaxation). Brown relaxation comes from the physical rotation of MNPs in the carrier fluid, the magnetic moment being locked onto the crystal anisotropy axis with a characteristic time, *τ*_B_, given in the herein Shliomis [41]:

(3) $$\tau_{B\;}=\;\frac{3\times \eta \times V_{\mathrm{hydro}}}{k\times T}$$

where, *η* is the viscosity of the carrier fluid, *k* the Boltzmann constant, *T* the temperature and *V*_hydro_ the hydrodynamic volume of the particle.

Neel relaxation is induced by the rotation of the MNP magnetic moment within the crystalline network, which occurs when the anisotropy energy barrier K.V (for a uniaxial crystal) is overcome. The released energy by the spin relaxation is dependent on the anisotropy energy of the MNP, i.e., the energy that imposes the direction of the magnetic moment within the crystalline network. The characteristic time, *τ*_N_, for Neel relaxation is written as follows,

(4) $$\tau_{N}{=\;\tau }_{0}\times {\;e}^{\frac{Ea}{k_{B}T}}$$

where *τ*_0_ is of the order of 10^−9^ s and *E*_a_ = *k*_B_×*T*, the thermal energy.

Our MNPs are composed of multiple single domain of maghemite cores aggregated inside a silica shell forming more or less well-defined short chain-like aggregates (see Appendix A for synthesis of core–shell nanoparticles γ-Fe_2_O_3_@SiO_2_ PEG/NH_2_). In liquids, both Neel and Brown relaxation occur; the predominant process being that with the shortest characteristic time (1/*τ*= 1/*τ*_N_ + 1/*τ*_B_). For a monodomain core, the time necessary to jump over the magnetic anisotropy energy barrier *K.V* is governed by Neel relaxation time with *τ*_N_, in the order of magnitude of a few nanoseconds. For bigger particles there is a contribution of Brown relaxation.

Fortin et al. determined by using different solvent viscosities (restriction of the particle orientation) that the loss process for maghemite particles with diameters below 16 nm in water was governed by Neel relaxation [42].

#### 3.1.3. Magnetic Hyperthermia on Chip

The device thus takes advantage of an optimal field (the field magnitude increases with the inverse of the gap size) and an increase of the reaction due to confinement. However, to fit into the 1 mm gap of the torus generating the field, the microfluidic chip’s thickness has to be less than 1 mm. This was achieved by using thin glass cover or by manufacturing ultra-thin PDMS layers (see Figure 1a,b). Two flow rates (0.11 and 0.01 µL s^−1^) within microfluidic channels (see Appendix B, Figure A3) were selected to investigate the magnetic nanoparticles residence time under the AC magnetic field and thus the time of DNA heating. Table 2 summarizes the experimental condition used in terms of flow rates, time of residence, volume of detection and time of experiment. Flow rates and time of experiment were calculated so that the final volume recovered after Δ*t* contains enough single-strand DNA to perform reliable fluorometric detection. The tore has a 1 cm² section. The path length under the tore where MNP are circulating in the serpentine configuration and is thus 1 cm. The residence time is given by Equation (5), as follows:

(5) $$\Delta t=\frac{w\times d\times h}{v}$$

where, Δ*t* is the residence time of the suspension of nanoparticles under the magnetic field, *w.d* the section of the channel under the electromagnet (*w*, the channel width, and *d*, the length under the magnet), *v* the flow rate and *h* the channel height.

DNA hybridization on core–shell nanoparticles γ-Fe_2_O_3_ @SiO_2_ PEG/NH_2_, release and target detection protocols are given in the Section 2.3.

**Figure 2:**
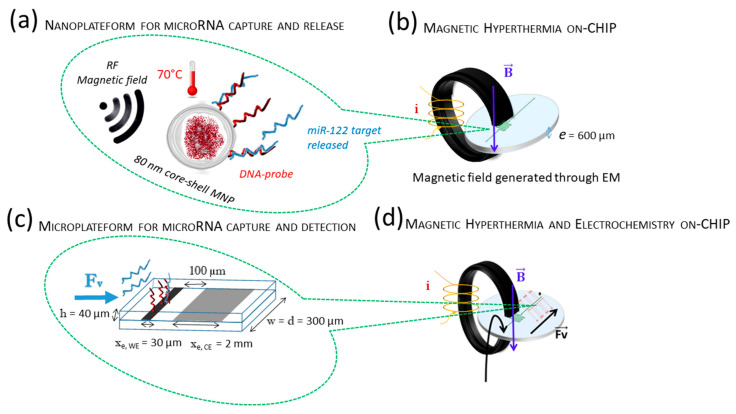
(**a**) Nanoplateform for microRNA release using functionalized core–shell magnetic nanoparticles (MNPs) and on-chip magnetic hyperthermia. (**b**) Schematic view of the localized magnetic hyperthermia through the ferrite gap. (**c**) Microplateform for microRNA capture and detection using functionalized a-CNx and on-chip electrochemistry, where WE stands for the working electrode, WE (width, w = 300 μm, length, xe = 30 μm), CE stands for the platinum counter electrode (w = 300 μm, xe = 2 mm) and the fluidic channel height and width are equal to 40 µm and 300 µm, respectively. (**d**) Schematic view of the device coupling magnetic hyperthermia and electrochemical detection. The vector Fv shown in (**c**,**d**) indicates the flow direction. The diameter of MNP and the length of microRNAs are not to the scale between them and together with the device dimensions.

**Figure 3:**
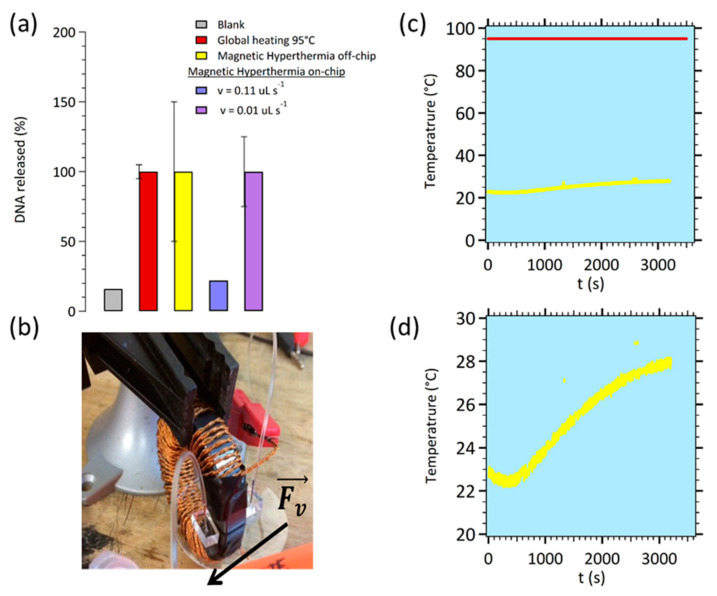
(**a**) Comparison of the results obtained for released DNA targets measured by fluorometric detection after: global heating (batch), off-chip magnetic hyperthermia (batch) and on-chip magnetic hyperthermia (according to the two flow rates used in geometry A). (**b**) Photography of the on-chip magnetic hyperthermia connected for samples introduction and collection (off-chip fluorometric dosing). (**c**) Monitoring of the temperature during the global heating 95 °C (red curve) compared to magnetic hyperthermia experiment (yellow curve). (**d**) Enlarged area of monitoring of the temperature from 23 to 28 °C during the magnetic hyperthermia experiment.

With Magnetic hyperthermia on-chip (Figure 2a,b) was carried out according to the two flow rates selected in geometry A (Figure A3b). Figure 3a shows the obtained results in terms of percentage of released DNA targets. To serve as reference, the maximum number of released DNA is calculated from a global heating at 95 °C for 20 min (that serves as a normalized curve reference i.e., 100%) and from off-chip magnetic hyperthermia to compare. “Global heating” means heating a 500 µL Eppendorf tube in a thermostatic bath. Blank measurements are carried out as controls without magnetic hyperthermia to quantify the baseline.

The fluorometric measurements and calibration are described above. Measured with a 0.11 µL s^−1^ flow rate, the amount of released DNA is slightly higher than the blank level range. The amount of released DNA obtained with an 0.01 µL s^−1^ optimized flow rate is found comparable both to global heating, and to off-chip magnetic hyperthermia. This proof of concept validates our proposed mechanism: an efficient heating at the vicinity of MNPs submitted to a homogeneous alternative magnetic field in the gap of the electromagnet. The heating induces a denaturation of double-stranded DNA, releasing the single-stranded target DNA collected at the outlet of the microfluidic channel. The difference between experiments with 0.11 and 0.01 µL s^−1^ flow rates is the time during which the MNPs are submitted to the alternative field. Indeed, the time needed for the denaturation of the DNA duplex (diffusion and zippering) is within a time scale of tens of seconds consistent with an increased release of target DNA with a lower flow rate and thus a longer residence time under AC field. These on-chip experiments exhibited efficient levels of DNA release at 28 °C (see Figure 3d), comparable to the off-chip setup but without the drawbacks of global heating at 95 °C (Figure 3c) as described in the introduction.

### 3.2. Coupling with Electrochemical Detection Methods

**Figure 4:**
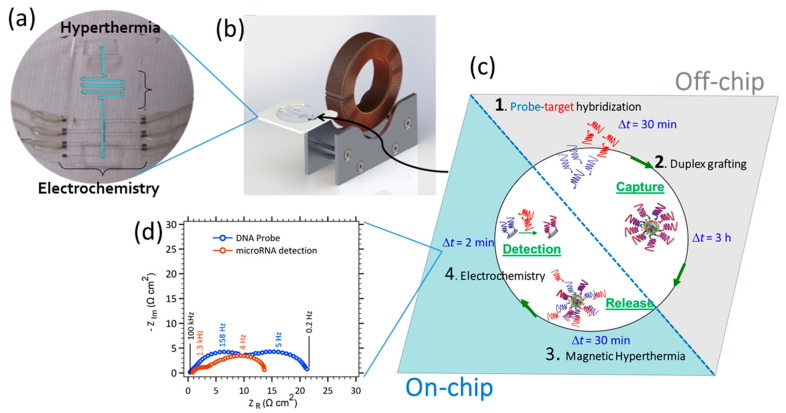
(**a**) Picture of the final PDMS/glass microfluidic setup for magnetic hyperthermia and electrochemical detection coupling. For convenient the microfluidic channel is highlighted in blue. (**b**) Microchip insertion for the localized magnetic hyperthermia through the ferrite gap. (**c**) On-chip hyperthermia protocol to release micro-RNA targets from magnetic nanoparticles coupled with electrochemical detection. (1) DNA probe and micro-RNA target are hybridized outside the chip. (2) Off-chip grafting of double-strand DNA/micro-RNA via peptide coupling on MNPs. (3) Introduction of functionalized MNPs in the microfluidic device for magnetic hyperthermia release under the AC magnetic field. Hybridization of released targets on the carbon microelectrode sensor functionalized with complementary probes. (4) micro-RNA electrochemical detection in 3 mM Fe^III^CN_6_^3−^/Fe^II^CN_6_^4−^, 10^−8^M methylene blue (MB) diluted in 0.5 M NaCl electrolyte. (**d**) Electrochemical impedance responses of the electrochemical on-chip detection of hybridization (following hyperthermia on-chip step) between 100 kHz and 0.2 Hz with 10 mV AC signal perturbation on the microchannel electrode sensor filled with of 3 mM [Fe(III)(CN)_6_]^3−^/[Fe(II)(CN)_6_]^4−^ + 10^−8^ M MB in 0.5 M NaCl. Nyquist plot of probe immobilized on carbon nitride microelectrode after circulating of 100 µg mL^−1^ DNA probe sequence diluted in 0.5 M NaCl for a 0.5 µL s^−1^ working flow (**○**). Target hybridization of miR-122 released for a working flow of 0.056 µL s^−1^ (**○**).

The whole PDMS/glass device that will be used in the following experiments is shown on Figure 4a. The network of microelectrodes, the microfluidic channel with the geometry B* (see Appendix B, Figure A3c), the thin PDMS cover and PDMS layer for inlet and outlet can be seen. The choice of an electrode pair set-up has been favored compared to a traditional three electrodes configuration for two main advantages. First, from a technological point of view, the microfabrication process contains less steps. For instance no planar Ag/AgCl reference electrode integration step in the microdevice is needed. Second, as described in Figure 2c and A3 caption (see Appendix B), the large CE (300 µm × 2 × 10^3^ µm) versus WE (300 µm × 30 µm) area ratio (A_CE_/A_WE_ ~ 70-fold) permits the drastic reduction of the CE current density (j_CE_ = i/A_CE_) and concomitantly stabilizes the rest potential at 0 volts with the addition of an equimolar mixture of the [Fe(III)(CN)_6_]^3−^/[Fe(II)(CN)_6_]^4−^ redox couple in solution. With these conditions, the CE electrode can be viewed as a pseudo-reference electrode [21,43]. In addition, to reducing the time of experiment, one way is to play on the geometry of the channel notably by increasing the volume under the AC field. The choice was made to increase the path length of the serpentine, giving rise to geometry B* with a serpentine of 5.7 cm in length instead of 1 cm (see Figure A3b versus Figure A3c). Table 3 shows the updated parameters.

A 0.01 µL s^−1^ flow rate was selected for geometry A to get a 12 s time of heating. In order to compare geometry A and B, time of heating (12 s) was kept constant leading to a 0.056 µL s^−1^ flow rate. The time of experiment Δ*t* was estimated to collect a 100 µL volume for fluorometric detection according to Equation (5). The calculation times were estimated at 2 h 48 min for geometry A and 30 min for geometry B*. Figure 4c describes the key steps of the modified protocol integrating electrochemical detectors for direct microARN detection without column-based purification (see Section 2.3. and Section 2.4.). To this goal, microelectrodes functionalization and electrochemical measurements protocol were described above (see Section 2.5).

## 4. Discussion

In our case, the magnetic hyperthermia can be tuned by either playing on the material (MNP core material (saturation magnetization and magnetic anisotropy), size, shape and interaction between the magnetic cores embedded in the silica) or by playing on the magnetic field (amplitude, frequency and waveform). The conversion of the dissipated magnetic energy into heat and temperature is complicated to model and measure (even though some orders of magnitude can be found in the literature, Yu et al. 2014 [44]). A very naive picture consists in considering that the specific loss power is proportional to the field amplitude squared (*H*_e_^2^) and to the frequency (*f*). For large fields, a better suited picture in terms of dynamic minor hysteresis loops would yield also an increase of the dissipated power when the field amplitude and frequency increase. In our setup, we demonstrated that the hyperthermia can thus be controlled as both the field intensity and frequency can be experimentally adjusted in microfluidics.

At this stage, we cannot really compare magnetic hyperthermia and a microheater since it is local heating without raising the temperature of the medium. Additionally, the measurement of the local temperature at the nanoparticles surface vicinity is not easy. It implies in depth modification of the structure of the nanoparticles. The denaturation of the DNA double strands and the detection of the target released is in itself indirect proof of the rise in temperature. Indeed, the melting temperature (Tm) of the double strand of DNA that we used in this work can be estimated around 55 °C. This value takes into account a sequence of 21 basis (7.14 nm in length), a 0.5 M saline buffer concentration and a 47.6% GC composition according to the method proposed by Meunier-Priest et al. [45]. In addition, by comparing Dias et al. [32] and Ge et al. [46] calculations, the melting temperature on the nanoparticle surface should approach the melting temperature in solution by 5–8 °C.

The advantage of on-chip electrochemical detection compared to fluorescence is highlighted in Figure 4. The limit of detection of such a device with microelectrodes in microfluidics with a quasi-static flow was previously found in the femtomolar range with gold microelectrodes [43], whereas it is in the nanomolar range for fluorometric detection. The channel geometry used was the one optimized (geometry B*) so that the MNPs were submitted to the magnetic field during 12 s (flow rate about 0.056 µL s^−1^). Carbon nitride microelectrodes were added to achieve robust covalent grafting [36], in contrast to using gold where the stability of the DNA probe layer is poor in time. Thus, contrary to fluorometric detection, where the solution must be retrieved at the outlet of the microfluidics channel, filtrated and diluted in cuvettes, the electrochemical detection is integrated in the downstream of the serpentine. As soon as target DNA strands are released due to the magnetic hyperthermia heating of the MNPs, they are captured by the probe-functionalized microelectrodes downstream. To this goal, the same flow rate is used for magnetic hyperthermia protocol and electrochemical detection. The protocol corresponding to the lowest 10^−16^ M LOD is the one for a hybridization step of 30 min, i.e., the time for which a sufficient amount of targets reached and hybridized on the microelectrode. This time of experiments can be decreased but it will be detrimental to LOD as the latter is flow dependent [43].

Magnetic hyperthermia coupled to on-chip electrochemical detection was performed according to the scheme depicted on Figure 4c, in a 30 min protocol (0.056 µL s^−1^ flow rate). Prior to that, on-chip carbon surface pretreatment had to be activated before the functionalization of electrodes according to the protocol described in the experimental section. The Nyquist plots highlight two time constants that are traditionally observed for reactive impedance mixed with diffusion impedance due to the redox reaction-diffusion at the microelectrodes. The experimental results are frequently simulated with a Randles equivalent circuit [47]. On Figure 4d, 21-base pairs of DNA molecules build a probe monolayer immobilized on the a-CNx microelectrode for detection in a one-step sequence of hyperthermia (target released from the MNPs) and electrochemical detection (target hybridization on functionalized a-CNx). The Nyquist plots between probe immobilization and target hybridization (flowing hyperthermia) show a decrease of charge transfer resistance (first loop) and the diffusion impedance (second loop) from 6.8 to 1.8 Ω cm² and from 13 to 10.3 Ω cm², respectively. It indicates the chemical hybridization of microRNA targets, the re-establishment of electron transfer due to the electrocatalytic and thus their detection. Indeed, in double stranded DNA, the base pairs are perfectly arranged in parallel plans with a partial overlap (pi-stacking). In a well-stacked DNA helix, it has already been proven that electron transfer kinetics are faster [48]. Single-stranded DNA are not as ordered. This difference in structure has been verified by measuring the optical density at 260 nm. The extinction coefficient of single stranded DNA is higher due to its lack of structure, and thus a lack of quenching due to the overlap [49]. In the working buffer, a specific DNA duplex redox intercalant, methylene blue (MB), was added. MB is an organic intercalant with a high k = 10^6^ M^−1^ affinity constant for DNA [50]. Its reduction is reversible with a 0 V redox potential (vs. NHE) lower than the one of Fe^III^(CN)_6_^3−^. During the electrocatalytic process through hybridized DNA, the electrons are transiting from the electrode to the intercalated MB and are accepted by the ferrocyanate in solution. The oxidized MB is again available for further electrochemical reductions. The Fe(CN)_6_^3−^ reduction promotes the complex diffusion along the helix, while the redox intercalant takes part in the electron transfer through the bases pi-stacking [51].

## 5. Conclusions

We demonstrated that integration of magnetic hyperthermia coupled to on-chip electrochemistry lead to a one-step microfluidic protocol including the release of short sequences of microRNA from DNA-modified superparamagnetic core–shell nanoparticles and their specific detection on DNA-modified carbon nitride microelectrodes. The upstream module is a transposition of magnetic hyperthermia in microfluidics, taking advantage of the high surface density of MNPs and the confinement of fluidics in nanovolumes. The hydrodynamic conditions were optimized according to the channel geometry. The MNPs were submitted to an AC field long enough for the duplex dehybridization and diffusion to take place efficiently. The downstream module performed the electrochemical detection with a set of microelectrodes. Optimized channel microelectrodes geometry allowed the fluidic volume to stay an optimal amount of time under the field, allowing the time of experiment to be decreased from several hours to 30 min. The combination of electrochemical detection and magnetic hyperthermia reduced time scales, volumes and allowed for the automation of some of the protocol steps. It opens the door for important developments (fundamental and application) in physics, chemistry and biology where a fine and dynamic tuning of a confined environment (concentrations, flow rates and time of residency of nanoparticles), where the microchannel volume of reaction is under the microliter, while maintaining the microchannel at room temperature.

## Figures and Tables

**Table 1:** Sequences of oligonucleotide strands.

Oligonucleotide	Sequence (5′ to 3′)
DNA Probe (P) for MNPs	5′-Carboxy C6-CAA ACA CCA TTG TCA CAC TGC-3′
DNA Probe (P’) for microelectrodes	5′-Amino C6-CAA ACA CCA TTG TCA CAC TGC-3′
Target (T)	5′-GC AGT GTG ACA ATG GTG TTT G-3′

**Table 2:** Comparison of optimized parameters for flow rates and residency time under AC field with fluidic geometry A (see Appendix B, Figure A3b) for on-chip magnetic hyperthermia and fluorometric detection.

Fluidic Geometry	v (µL s^−1^)	Residence Time (*t*)	Volume of Detection (µL)	Δ*t* ^§^
A	0.11	3 s	400	2 h 48 min
A	0.01	12 s	100	2 h 48 min

^§^ minimum time of experiment to get a sufficient volume *V* for fluorometric detection.

**Table 3:** Comparison of optimized parameters depending on the different geometries of the fluidic channel for on-chip magnetic hyperthermia and fluorometric detection.

Fluidic Geometry	*V*^#^ (µL s^−1^)	Δ*t* ^§^	Sensitivity (M)
Geometry A	0.01	2 h 48 min	10^−8^
Geometry B *	0.056	30 min	10^−8^
Geometry B *	0.056	30 min	<10^−16^

^#^ Maximum selected flow rate so that DNA undergo a T temperature during 15 s. ^§^ Minimal measured time of experiment to get a sufficient volume V (100 µL) for fluorometric detection.* See configuration in Figure A3c.

**Figure A1:**
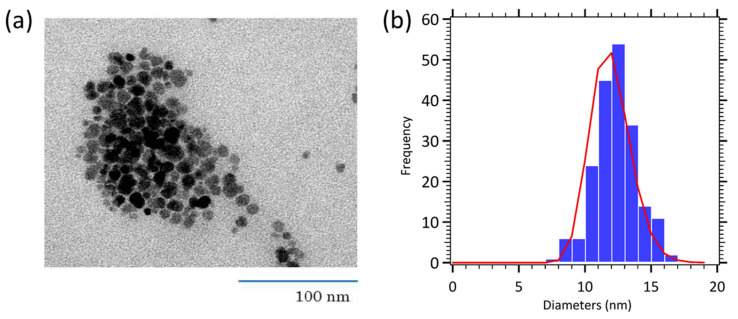
TEM image (**a**) and size distribution (**b**) of size sorted γ-Fe_2_O_3_ nanoparticles.

**Figure A2:**
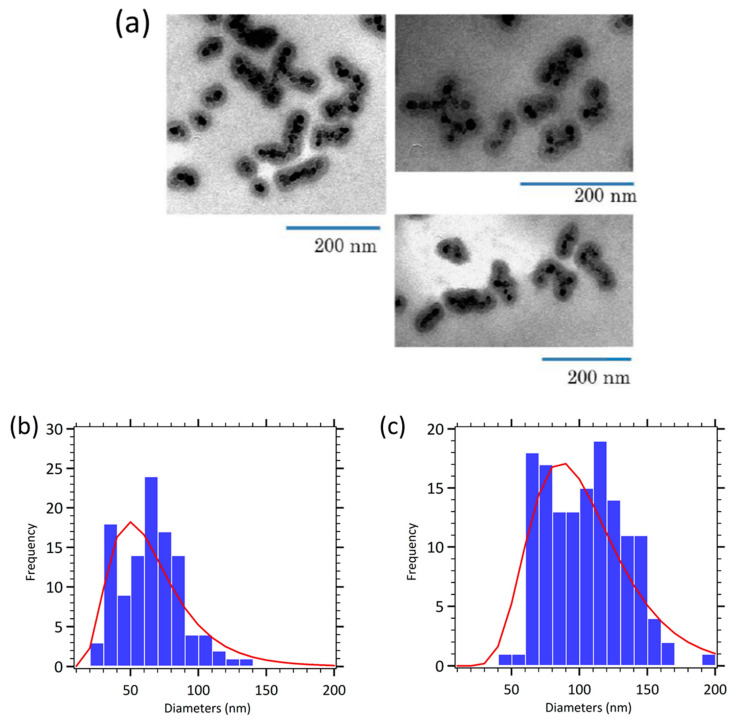
TEM images (**a**) and size distribution: (**b**) cores and (**c**) core–shell nanoparticles of core–shell nanoparticles γ-Fe_2_O_3_ @SiO_2_ PEG/NH_2_.

## Data Availability

Data sharing is not applicable to this article.

## Appendix A

### Synthesis of Core–Shell Nanoparticles γ-Fe_2_O_3_@SiO_2_ PEG/NH_2_

The size sorted maghemite γ-Fe_2_O_3_ nanoparticles were obtained by a Massart’s coprecipitation method [52]. One liter of NH_3_ 22.5% was added to an acidic iron (II) and iron (III) ions solution (180 g of FeCl_2_, 100 mL of HCl 37% and 715 mL of FeCl_3_ 35.2%, in 3.5 L of distilled water) under agitation. After rinsing with distilled water using magnetic decantation, the obtained Fe3O4 nanoparticles were oxidized in maghemite nanoparticles and then size sorted and citrated due to previously reported protocols [53,54]. Size distribution of sorted γ-Fe_2_O_3_ nanoparticles was characterized by transmission electron microscopy (TEM) on a JEOL 1011 instrument (Figure A1a). Nanoparticles’ images analysis was done with Image J software on 200 nanoparticles using a spherical model. The size distribution (Figure A1b) was analyzed as a Log Normal distribution leading to a mean physical diameter of 11.8 nm (σ = 0.12).

Silica coating was performed using a modified previously reported protocol [55,56,57]. Of size sorted γ-Fe_2_O_3_ nanoparticles solution ([Fe] = 1.02 M) 700 µL were diluted in 50 mL of water and 100 mL of ethanol (99%). Then 6 mL of NH_4_Cl solution (0.1 M) was added in order to increase the ionic strength thus inducing slight aggregation of the magnetic cores. Subsequently, 900 µL of TEOS and 1.88 mL of NH_3_ 30% were added. After 2 h of agitation, the functionalization of the silica shell by short polyethylene glycol (PEG) chains and amine functions was carried out by the addition of 300 µL of TEOS, 350 µL of PEOS and 150 µL of APTS. The mixture was stirred overnight. The resulting nanoparticles were then rinsed 3 times with a mixture of diethylether/ethanol 15:1 and finally redispersed in 50 mL of a 3-morpholinopropane-1-sulfonic acid (MOPS) buffer at 0.1 mol/L and pH = 7.4.

The obtained core–shell nanoparticles γ-Fe_2_O_3_ @SiO_2_ PEG/NH_2_ were characterized by TEM (Figure A2a). The nanoparticles’ image analysis was done with Image J software using an elliptical model on 110 nanoparticles. The size distributions were analyzed as Log Normal distributions, leading to a mean major axis of 60.3 nm (σ = 0.44) for the magnetic cores (Figure A2b) and of 97.9 nm (σ = 0.35) for the core–shell nanoparticles (Figure A2c).

In comparison to spherical core shell nanoparticles previously described [56] larger core shell nanoparticles with an elliptical morphology are obtained. Size sorted γ-Fe_2_O_3_ nanoparticles form short chain-like structures inside the silica shell. This can be explained by the increase of the ionic strength, leading to a slight aggregation and an alignment of magnetic nanoparticles prior to the silica coating.

Thus preaggregation of γ-Fe_2_O_3_ nanoparticles seems to allow increasing the size of the magnetic cores, and modifying the morphology from a spherical to an elliptical one, although TEM pictures show some polydispersity, resulting in larger size distribution for the cores and the core shell nanoparticles.

## Appendix B

### Photography and L-Edit Channel and Microelectrodes Geometries

As displayed on Figure A3, all lithographic masks for microelectrodes and channels were designed using L-Edit software (Tanner EDA). Briefly, the microelectrodes were prepared on glass slides (first cleaned in a piranha solution (H_2_SO_4_/H_2_O_2_, 1:1) and rinsed in water, acetone, isopropanol and dried with nitrogen. The AZ-5214 photoresist was spin-coated (30 s, 1000 rpm, 250 rpm s^−1^) and baked-up for 90 s using a hot plate (125 °C). Subsequently, the photoresist was exposed to UV light (10 mW, MicroTec MJB4) baked-up for 90 s, flood exposed for 30 s and developed for 45 s. Afterwards, the slides were rinsed in water, and cleaned by using reactive ion etching (RIE Nextral NE100, 180 s, 50% O2, 10 W) to remove all resist residues. An evaporator (Pfeiffer Vacuum Classic 500) was used to deposit a 5 nm titanium layer for contact and 150 nm of platinum followed by a classic lift-off process in acetone. This process was replicated for a third layer of amorphous carbon nitride a-CNx, x = 0.12 (stoichiometry C/N ratio checked in XPS), 220 nm (thickness checked in SEM) deposited with DC magnetron sputtering (MP300s Plassys, t = 20 min, 200 W, P < 2 × 10^−7^ Torr) with a graphite target (ϕ = 75 mm, e = 5 mm, 99.99% of purity), under a flow of nitrogen (N2 = 3%, PAr + N2 = 0.4 Pa). The PDMS serpentine and the glass substrates with the a-CNx microelectrodes were washed with isopropanol and dried with nitrogen. The electrode slides and the PDMS microchannel were activated with a plasma cleaner (Nanonex Ultra-100, 60 s, 100% O2, 20 W) to favor adhesion, and were mounted together. The electrode alignment on the microfluidic channel was done manually with a binocular. A heat treatment step for 15 min (95 °C) followed. For the electrical connections to the electrochemical equipment, wires were soldered onto the platinum electrodes. The microfluidics devices have several sets of working (WE, CE) couples. The main steps of the microfluidic device fabrication are summarized on Figure A3.
